# *Staphylococcus aureus* and Hyper-IgE Syndrome

**DOI:** 10.3390/ijms21239152

**Published:** 2020-12-01

**Authors:** Bonggoo Park, George Y. Liu

**Affiliations:** 1 Division of Pediatric Infectious Diseases and the Immunobiology Research Institute, Cedars Sinai Medical Center, Los Angeles, CA 90048, USA; parkbg35@gmail.com; 2 Department of Pediatrics, University of California San Diego, La Jolla, CA 92093, USA

**Keywords:** hyper-immunoglobulin E syndrome (HIES), primary immunodeficiency disease, *Staphylococcus aureus*, signal transducer and activator of transcription 3, T helper 17 (T_H_17) cell, chemokines, antimicrobial peptides, staphylococcal lung and skin infections

## Abstract

Hyper-immunoglobulin E syndrome (HIES) is a primary immunodeficiency disease characterized by recurrent *Staphylococcus aureus* (*S. aureus*) infections, eczema, skeletal abnormalities and high titers of serum immunoglobulin E. Although the genetic basis of HIES was not known for almost a half century, HIES most frequently exhibits autosomal dominant trait that is transmitted with variable expressivity. Careful genetic studies in recent years identified dominant-negative mutations in human signal transducer and activator of transcription 3 (*STAT3*) gene as the cause of sporadic and dominant forms of HIES. The *STAT3* mutations were localized to DNA-binding, SRC homology 2 (SH2) and transactivating domains and disrupted T helper 17 (T_H_17) cell differentiation and downstream expression of T_H_17 cytokines IL-17 and IL-22. Deficiency of IL-17 and IL-22 in turn is responsible for suboptimal expression of anti-staphylococcal host factors, such as neutrophil-recruiting chemokines and antimicrobial peptides, by human keratinocytes and bronchial epithelial cells. T_H_17 cytokines deficiency thereby explains the recurrent staphylococcal lung and skin infections of HIES patients.

## 1. Introduction

*S. aureus* causes a wide range of pathological conditions ranging from minor skin and soft tissue infections to life-threatening invasive diseases [1]. Methicillin-resistant *S. aureus* (MRSA) strains are particularly problematic since they cause persistent colonization of mucosal tissues in at least 20% of individuals, and thereby predispose the colonized subjects to infections and spread of their isolates to close contacts [2]. Staphylococci use many strategies to evade host innate and adaptive immune defenses and survive hostile environments [1,3,4,5,6]. These strategies include resistance to specific antimicrobial peptides, neutralization of reactive oxygen species (ROS), inactivation of complement, inhibition of neutrophil migration, and evasion of phagocytosis [1,7]. MRSA is recognized as a more formidable pathogen than most other bacteria, and their proliferation within healthcare and community settings has contributed to the current public health crisis [8,9,10]. Combating MRSA has necessitated routine use of vancomycin and other last line agents for treatment of *S. aureus* infections [11,12]. The dire circumstance of increased disease burden along with spread of antibiotic resistance has prompted public health officials and scientists alike to look for novel therapeutic approaches. Among these, vaccination has long been the Holy Grail since it has the potential to effectively address both disease burden and overuse of antibiotics. Although more than ten passive and active staphylococcal vaccines have been tested in clinical trials in the past decades, all vaccines have been found to be ineffective in humans without a clear explanation [13]. Useful insight on alternative staphylococcal vaccine strategies and host adaptive immune responses to *S. aureus* have come from studies of a particular immunodeficiency condition, hyper-IgE syndrome (HIES), which confers susceptibility to recurrent *S. aureus* infections. Below we will review our current understanding of HIES in relation to *S. aureus* infections and will refer readers to other excellent reviews for overview of *S. aureus* pathogenesis [1,3,4,5,6]. 

## 2. Hyper-IgE Syndrome (HIES)

Hyper-IgE syndrome (HIES), also called Job’s syndrome or Buckley’s syndrome, was first described as a rare primary immunodeficiency disease characterized by recurrent staphylococcal “cold” skin and pulmonary abscesses, eczematoid dermatitis, markedly elevated levels of serum IgE and eosinophilia, reduced neutrophil chemotaxis, and variably impaired T cell function [14,15]. 

### 2.1. Genetics

Job’s syndrome was first described in 1966 and recognized as hyper-IgE syndrome (HIES) in 1972 on the basis of extreme elevation of IgE in patients with the syndrome. The involvement of multiple organs including various skeletal malformations was delineated over several decades [14,15,16]. However, only recently was significant progress made in the molecular understanding of both clinical phenotype and pathogenesis of the autosomal dominant form of HIES (AD-HIES). Although mutations in dedicator of cytokinesis 8 (*DOCK8*) gene is responsible for the autosomal recessive form of HIES, this review will focus on the more common and better characterized form of HIES caused by *STAT3* mutations.

**Figure 1:**
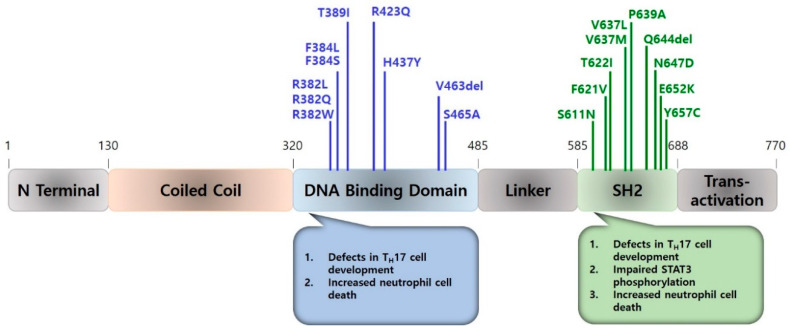
Schematic structure of mutations in *STAT3*. Previously described mutations, which are likely involved in recurrent *S. aureus* infections in HIES patients, are shown in the upper part of the figure. The lower part of the figure described the relevant abnormalities involved in staphylococcal infections of HIES patients.

Early research on the genetics of HIES benefited from the discovery of a supernumerary marker chromosome in a patient with sporadic HIES. Microdissection and fluorescence in situ hybridization (FISH) analysis of lymphocytes and skin fibroblasts from the patient identified a small interstitial deletion of one homologue of chromosome 4q21 [17] and pointed to the genes disrupted or lost from the marker chromosome as possible causes of HIES phenotypes [17]. In a subsequent study, 19 kindreds with multiple cases of HIES were recruited, scored based on clinical and laboratory findings, and genotyped with polymorphic markers in the candidate region on human chromosome 4. The linkage and multipoint analyses and simulation testing corroborated the earlier finding that the proximal 4q region harbors the disease locus for HIES [18]. *STAT3* mutations were eventually identified as the cause of autosomal dominant HIES in 2007, wherein the mutated *STAT3* inhibited the activity of the wild-type allele while homozygous *STAT3* mutations led to lethality [19,20,21,22]. The identified *STAT3* mutations were shown to be largely missense or in-frame deletions located primarily in the DNA binding and SH2 domains (Figure 1) [19,20,23,24,25]. Although non-immunologic features such as high palate, increased inter-alar distance, and increased scoliosis appeared to be linked to mutations in the SH2 domain, patients with mutations in the DNA binding and SH2 domains generally had similar phenotypic features [26]. 

STAT3 is a signaling molecule that functions downstream of several cytokine receptors including IL-6R, IL-IL-11R and IL-21R [27,28]. Patients with IL-11RA deficiency have craniosynostosis, maxillary hypoplasia, dental abnormalities, but show no immunodeficiency [29]. Subjects with IL-6R deficiency present with recurrent skin and lung infections, eczema, high IgE levels, and eosinophilia [30], whereas patients with IL-21R deficiency suffer from recurrent respiratory tract infections and exhibit impaired B cell proliferation, immunoglobulin class-switch and reduced T cell effector function [31]. Consistent with the expected pleiotropic effect of *STAT3* mutations on clinical phenotypes, STAT3-HIES patients exhibit both immune and non-immune phenotypes that could be attributable to defective signaling through at least IL-6R, IL-11R, and IL-21R.

### 2.2. Immunoglobulins 

The discovery of *STAT3* mutations as the immunologic basis of autosomal dominant HIES paved the way towards unraveling the mechanism for reduced neutrophil chemotaxis and variable T cell defects in HIES, but also towards the etiology behind hyper-IgE and abnormal humoral responses in HIES (ref) [16,32,33]. To define candidate genes involved in the pathophysiology of HIES, investigators interrogated *S. aureus*-stimulated CD4+ T cells and peripheral blood mononuclear cells from HIES and control subjects, and showed significantly greater numbers of lysosome- and immunoglobulin-related genes that were up-regulated in HIES patients compared to controls [34]. However, when HIES patients were tested for antibody responses to bacteriophage φX174 (φX174) and several protein and polysaccharide vaccines, responses were heterologous, with many of the subjects showing low level or accelerated decline of antibody levels, with failure to class switch from IgM to IgG in others, consistent with suboptimal humoral responses that could confer susceptibility to infections in these patients [35]. Another study of HIES patients noted markedly elevated levels of serum IgE to *S. aureus* and *Candida albicans* (*C. albicans*), compared to IgE from normal subjects with parasitic infections [36]. Because IgE antibodies from HIES patients did not bind other common pathogens, the specific IgE antibodies were proposed to actually confer susceptibility to *S. aureus* and *C. albicans* infections [36]. Investigation of other antibody isotypes also showed elevated serum anti-*S. aureus* IgM, lower than expected level of anti-*S. aureus* IgG given the IgM level, and a deficit in the level of anti-*S. aureus* IgA in HIES patients [37]. When analyzed against the incidence of infections at mucosal surfaces and adjacent lymph nodes, an inverse correlation was shown to the level of serum anti-*S. aureus* IgA and IgE, and total serum IgE and IgD, thus pointing to regulatory defects in humoral responses in HIES patients that likely contributed to staphylococcal susceptibility [37]. 

As discussed, STAT3 acts as a signaling molecule downstream of IL-21R [27]. IL-21 produced by follicular T helper cells confers B cell help by activating STAT3 in germinal centers and inducing immunoglobulin class switching and maintaining high titer and high affinity antigen-specific antibody response [38,39]. Uniquely, IL-21 suppress antigen-induced IgE production by inducing apoptosis of B cells committed to produce IgE and by inhibiting germline C(epsilon) transcription in IL-4 stimulated B cells [40,41]. Consistent with these mechanisms, STAT3-HIES patients have reduced plasmablast and memory B cell compartment but have significant numbers of IgE plasma cells in bone marrow and lymph nodes [42]. HIES B cells exhibit suboptimal proliferative response to IL-21 and reduced immunoglobulin heavy chain variable region gene somatic hypermutation [42]. 

In agreement with humoral immunodeficiency, administration of replacement immunoglobulin to STAT3-HIES patients with low level of anti-*S. aureus* IgG increased serum *S. aureus*-specific IgG and improved the course of staphylococcal infection [43]. 

### 2.3. TLRs

Abnormalities in specific immune responses of HIES patients led investigators initially to hypothesize that defects in Toll-like receptor (TLR) signaling pathways underlie the primary immunodeficiency. In studies stimulating blood samples from HIES patients with Toll-like receptor (TLR) pathway agonists, although HIES individuals showed an interferon-γ (IFN-γ) secretion defect in T cells following phorbol 12-myristate 13-acetate (PMA) challenge, they produced normal levels of several TLR-regulated proinflammatory cytokines and showed no unique mutations or polymorphisms in several TLR pathway genes [44,45]. These studies confirmed an imbalance in T cell responses in HIES patients but gave no indication of defects in toll-like receptor signaling.

### 2.4. STAT3

Following analysis of cytogenetic and linkage data that narrowed HIES gene linkage to a locus on chromosome 4q [18], subsequent investigations of HIES patients and their families identified *STAT3* gene as a candidate gene from measurements of cytokine secretions from stimulated leukocytes and gene expression profiles in resting and stimulated cells [19]. Although homozygous recessive tyrosine kinase 2 (TYK) deficiency also can present with primary immunodeficiency with mild IgE elevation, *STAT3* mutations are the predominant cause of sporadic and familial hyper-IgE syndrome [20,46]. Missense mutations and single codon in-frame deletions were identified in *STAT3* genes in familial and sporadic cases of the hyper-IgE syndrome, where discrete mutations included hot spots in the DNA binding and SH2 domains [19]. Investigation of peripheral blood cells from HIES patients unveiled defective response to selective cytokines IL-6 and IL-10, decreased STAT3 binding to DNA in those cells, along with dominant-negative effects of the non-functional STAT3 in presence of wild-type STAT3. Altogether, the findings underline the complex and critical role played by STAT3 in the signaling of multiple cytokine pathways and which are defective in HIES [20]. 

For control of *S. aureus* infection in the lungs and skin, studies have identified several critical roles played by STAT3. In pneumonia, clearance of *S. aureus* requires the activation and robust phosphorylation of STAT3 in lung epithelium [47], which in turn regulate expression of Reg3y (regenerating islet-derived 3y) through direct binding to the *Reg3γ* promoter [47]. Reg3γ is an antimicrobial peptide that binds and induce dose-dependent killing of *S. aureus* [47]. During skin infection, IL-17A and IL-22 produced by activated T cells induce keratinocytes to express antimicrobial factors CXCL8 and β-defensin 2 and 3 (BD-2 and -3), which critically control *S. aureus* infection. Thus, mutations in *STAT3* leading to defective T cell development affect the production of antimicrobials required to control *S. aureus* [48]. T_H_17 cells play a central role in host immunity on mucosal surfaces against extracellular bacterial infections, by producing the T_H_17-related cytokines IL-22 and IL-17A that regulate chemokines and granulocyte colony–stimulating factor production in the lung. IL-22 further increases lung epithelial cell proliferation and transepithelial resistance to injury [49,50]. IL-17 and also IL-1β promote neutrophil recruitment to the site of *S. aureus* infection in the skin to induce bacterial clearance [50]. A study showed that the presence of eosinophils reduces the half-life of STAT3-deficient neutrophils ex vivo, although no specific aberrations in these neutrophil functions were detected [51]. Infection of the STAT3-deficient neutrophils in presence of eosinophils, thus accelerated neutrophil cell death and had the potential to negatively impact pathogen burden and tissue pathology in the HIES subjects [51]. Another investigation sought to determine the cutaneous response of HIES and normal subjects after induction of suction blisters and challenge of the wound with lethally irradiated *S. aureus*. Interestingly, no difference in cutaneous production in IL-17A or IL-17F was observed in the two groups albeit the time course of bacterial challenge was short. HIES patients however produced more TNF-α that was associated with reduced IL-10 family signaling in the blister infiltrating cells and defective epithelial cell function, findings that were recapitulated in a mouse model of HIES and shown to involve defective epithelial to mesenchymal transition (EMT) [52]. 

## 3. T_H_17 Cells in Staphylococcus Infections of Hyper-IgE Syndrome (HIES)

### 3.1. T_H_17 Cells

T_H_17 cells are a helper T cell subset distinct from T_H_1, T_H_2 or regulatory T cells that requires unique cytokines and transcription factors for their differentiation. T_H_17 cells produce so-called T_H_17 cytokines including IL-17A, IL-17F and IL-22 that protect the host from infections by various microorganisms [53,54,55] but are important immunologic players or drivers of various immunological disorders. One particularly crucial role of T_H_17 cells is their defense against extracellular pathogens which are not effectively cleared by T_H_1- and Th2-type immune responses. Mice that produce neither IL-17A nor IL-17F are susceptible to skin infection by *S. aureus* [56], since T_H_17-type cytokines IL-17A and IL-17F critically recruit neutrophils to clear *S. aureus* [57]. IL-22 which activates keratinocyte production of antimicrobial peptides, BD-2 and -3 via STAT3, is essential for defense against Gram-negative bacteria, as evidenced by susceptibility of IL-22 deficient mice to lung infections caused by *Klebsiella pneumoniae* and *Mycoplasma pneumoniae* [49,58,59]. Deficiency of IL-17A receptor or administration of anti–IL-17A neutralizing antibodies impairs granulopoiesis and mobilization of neutrophils against systemic infection induced by *C. albicans* [60]. These findings indicate that T_H_17 cells play a key role in immune responses to extracellular bacteria and fungi in mice. 

Differentiation of T_H_2 from naïve CD4+ T cells and T_H_2 cell secretion of IL-5 and IL-13 appear normal in STAT3 patients [61]. However, IL-5 and IL-13 levels in PBMC are elevated in STAT3 patients compared with control subjects, suggesting that cells other than CD4+ T cells in PBMC are responsible for the increased T_H_2 cytokine production [61]. When treated with IL-10, DC isolated from STAT3 patients show impaired up-regulation of surface inhibitory molecules PD-L1 and ILT-4, and reduced ability to induce naive CD4+ T cell differentiation to FOXP3+ induced Treg cells (iTreg cells), both of which could contribute to the inflammatory changes in HIES [61]. 

### 3.2. STAT3 Mutations

Studies have shown that autosomal dominant-negative mutations in the human *STAT3* gene are largely responsible classical multisystem HIES [19,20,23]. Most mutations of the *STAT3* gene in HIES patients are localized to the DNA binding and SH2 domains, with few mutations shown in the transactivation domain [20,23]. The mutants were non-functional on their own and exhibited dominant-negative effects in the presence of wild-type STAT3 [20]. *STAT3* mutations lead to impaired T_H_17 cell development, with SH2 domain mutations showing reduced STAT3 phosphorylation, and mutations in the DNA binding domain exhibiting STAT3 phosphorylation comparable to normal controls [23]. Broadly, there are two types of HIES. Type 1 HIES displays abnormalities in multiple organ systems, including the skeletal, dental and immune systems that are related to the mutations in *STAT3*, whereas a null mutation in tyrosine kinase 2 (*TYK2*) causes type 2 autosomal recessive form of HIES with abnormalities confined to the immune system [62,63,64,65]. 

Analyses of cytokine responses in both types of HIES showed severe defects in signal transduction that lead to depressed IL-6 and IL-23 expression and impaired T_H_17 differentiation [62,63,65]. Thus, T cells from HIES individuals produce IL-2 and IFN-γ but not IL-17 on stimulation with staphylococcal enterotoxin B or *C. albicans* [66]. CD4 + T cells derived from HIES subjects were unable to generate T_H_17 cells in vitro and in vivo because of low retinoid-related orphan receptor (ROR)-γt expression [24,25,66]. As T_H_17 cells are important for the clearance of fungal and extracellular bacterial infections, the inability to produce T_H_17 cells appears to be the primary mechanism responsible for the susceptibility of HIES patients to the recurrent infections [24,25,60,67].

### 3.3. Molecular Mechanisms Conferring Site-Specific Susceptibility to S. aureus Infections in HIES Patients

**Figure 2:**
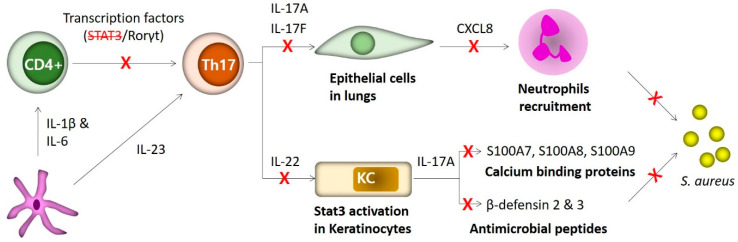
In HIES patients, *STAT3* mutations cause defects in T_H_17 cell differentiation and in host defense against *S. aureus* infection. In normal subjects, IL-1β and IL-6, secreted by dendritic cells (DCs), result in the differentiation of T_H_17 cells from CD4+ T cells through activating its transcription factor retinoic acid-related RORγt in STAT3-dependent way. Then, in presence of IL-23, T_H_17 cells secrete IL-17A and IL17F stimulating epithelial cells in lungs for production of chemokines such as CXCL8 to recruit polymorphonuclear leukocytes (PMNs) for phagocytic killing of *S. aureus*. IL-22 out of T_H_17 cells also activates STAT3 in keratinocytes to produce β-defensins and calcium binding proteins along with IL-17A to defend against extracellular *S. aureus*. In HIES patients, mutations in *STAT3* result in failure of T_H_17 differentiation, which in turn leads to the inability of epithelial cells to produce CXCL8 for recruitment of neutrophils and of keratinocytes to trigger production of β-defensins and calcium binding proteins to kill *S. aureus*.

HIES patients suffer from recurrent fungal and *S. aureus* infections most likely from defective T_H_17 cell differentiation [23,24,25,66]. Staphylococcal infections in HIES patients are often confined to the skin and lungs, in comparison to patients with neutrophil deficiency who suffer from infections occurring in a wide variety of organs including the lungs, lymph nodes, liver, bone, gastrointestinal tract, kidney, and brain [68]. To explain the predilection of HIES infections for the lungs and skin, investigators compared expression of chemokines and antimicrobial peptides in a variety of immune and non-immune cells. The study showed that keratinocytes (skin) and bronchial epithelial cells (lungs) require both T_H_17 cytokines and classical proinflammatory cytokines, acting in synergy, to produce the anti-staphylococcal factors. In comparison, other cell types, including fibroblasts, endothelial cells and macrophages, require only the common proinflammatory cytokines to produce the anti-staphylococcal factors [48,62]. Compared to T cells derived from normal subjects, HIES T cells have defects in T_H_17 cytokines, IL-17A and IL-22 production, but show normal levels of common cytokines IL-1β, TNF-α and IFN-γ (Figure 2) [48]. Altogether, these findings explain the predisposition of HIES subject to infections of the lungs and skin, but not of other organ systems [48]. Accordingly, the local application of neutrophil-recruiting chemokines and β-defensins or other antimicrobial peptides could be as a strategy to treat recurrent skin- and lung-restricted staphylococcal infections in HIES patients. 

### 3.4. Antimicrobial Peptides in Dermatitis of HIES Patients

IL-17 and IL-22, the effector cytokines produced by T_H_17 cells, regulate the production of antimicrobial peptides that directly kill microbes in the gut and lungs. Antimicrobial proteins can be constitutively expressed, but some antimicrobials in skin epithelial cells and on mucosal surfaces can be induced by cytokines secreted by T_H_17 cells, and thus underline the importance of this helper T-cell lineage in skin and mucosal immunity [69,70,71]. Microarray gene expression analysis have shown that IL-17 is the most potent cytokine to induce human β-defensin 2 (hBD-2) expression in primary human airway epithelial cells. The IL-17R-mediated signaling pathway that stimulates hBD-2 gene transcription in response to microbial infection in airway epithelium involves Janus kinase (JAK) and nuclear factor-κB (NF- κB) [72,73,74]. Interestingly, neither resting nor activated immune cells express IL-22 receptor and therefore do not respond to IL-22 stimulation in vitro and in vivo. In keratinocytes, upregulation of IL-22 receptor occurs upon IFN-γ stimulation, which allows IL-22 to activates STAT3 and increases β-defensin 2 and β-defensin 3 expression [75].

Skin diseases such as psoriasis and atopic dermatitis have been linked to abnormal production of antimicrobial peptides in dermal pathology [76,77]. Dermal concentration of antimicrobial peptides increases during inflammatory conditions and is usually negligible in normal skins [78]. Immunohistochemical study of human skin-biopsy samples shows significantly lower concentration of cathelicidins (LL-37) and β-defensins in patients with acute and chronic atopic dermatitis, but high amount of the peptides in psoriasis patients. Associated with low antimicrobial peptide expression, atopic dermatitis patients have more frequent *S. aureus* colonization and infection of the skin lesions. LL-37 activity appears to synergize with hBD-2 anti-staphylococcal activity; therefore, a deficiency of these antimicrobial peptides may account for the susceptibility of atopic dermatitis patients to *S. aureus* skin infections [76,77,79].

## 4. Conclusions

Genetic and immunologic characterization of HIES has been one of the more remarkable scientific stories in the study of human primary immunodeficiencies. HIES started as a constellation of discrete clinical and laboratory findings, which were linked by specific genetic defects. The genetic discoveries in turn led to the identification of disrupted immune pathways that explained enhanced infectious susceptibility of HIES patients. Because HIES has variable clinical manifestations, single laboratory index or clinical feature remain insufficient to secure diagnosis of the disease. Likewise, supportive and prophylactic skin care and antibiotics remain the most effective treatment to date; but the genetic and immunologic advances in the pathogenesis of HIES promise to usher in novel therapeutic approaches in the treatment of chronic mucocutaneous candidiasis and staphylococcal infections.

Although investigations have focused primarily on the T_H_17 axis and downstream effector functions, many aspects of HIES immune and non-immune defects, including IL11R and other related mechanisms underlying common manifestations such as scoliosis, eczema, aneurysms, atherosclerosis, and osteoporosis remain to be more fully elucidated. Reports of abnormal anti-*S. aureus* antibodies in sera and saliva of HIES patients along with suboptimal memory responses to antigens point to defects in IL21R signaling that explain aspects of the humoral immune response of HIES patients.

*STAT3* mutations have been identified as important causes of multisystem HIES, adding to previous finding of *TYK2* mutations as causes of HIES, and suggest complex cytokine signaling defects to be at the heart of HIES pathogenesis. Discovery of *STAT3* mutations as the genetic basis of major HIES will facilitate early and definitive diagnosis in spite of paucity of specific clinical features in many patients and will permit timely administration of prophylactic antibiotic. *STAT3* mutations disrupt T_H_17 generation in vitro and in vivo and highlight the importance of STAT3 in T_H_17 differentiation and T_H_17 effector role in infection of HIES patients and other immune-mediated diseases. Conversely, the importance of T_H_17 and related cytokines in combating *S. aureus* cutaneous infections is well documented for HIES patients but also for normal subjects [50]. Thus, increasing production of IL-17 or T_H_17 related cytokines constitutes an approach to treat infections in HIES and normal subjects.

For HIES patients, although T cells exhibit defects in production of T_H_17 cytokines such as IL-17 or IL-22, production of other proinflammatory cytokines including IL-1β and TNF-α is normal upon challenge with staphylococcal antigens. The defective cytokine production selectively impacts anti-staphylococcal defense in keratinocytes and bronchial epithelial cells but not other cell types, and thus provides a possible molecular explanation for the selective susceptibility of HIES patients to skin- and lung-infections, but not systemic staphylococcal infections. Accordingly, IL-17 or IL-22 represent potential local therapeutic approaches for treatment of infections in HIES patients. Beyond HIES patients, *S. aureus* infections remain one of the most frequent causes of infectious morbidity and mortality worldwide. With the universal failure of antibody-based strategies against *S. aureus* in human vaccine trials, an immunization approach that promotes anti-staphylococcal T_H_17 development, as supported by HIES findings and several recent studies, could represent an effective strategy to addressing the failed vaccines [80,81].
